# Preface

**Published:** 2008

The Jan-Dec 2008 issue of *MSM* is a theme issue on Medicine, Mental Health, Science, Religion and Well-being.

## Editorials

**C. Robert Cloninger,** M.D. Wallace Renard Professor of Psychiatry, Genetics, and Psychology; Director, Sansone Center for Well-Being, writes an editorial, ‘On Well-Being: Current Research Trends and Future Directions’ [p3–9].

**K.W.M. Fulford,** DPhil, FRCP, FRCPsych. Professor of Philosophy and Mental Health, University of Warwick and Member of the Philosophy Faculty, University of Oxford, writes another editorial, ‘Values-Based Practice: A New Partner To Evidence-Based Practice And A First For Psychiatry?’ [p10–21].

**Peter Q. Eichacker** M.D. **and Charles Natanson** M.D. from the Critical Care Medicine Department, Clinical Center, National Institutes of Health, Bethesda, MD 20892, USA write the third editorial, ‘Medical guidelines and performance measures: The need to keep them free of industry influence’ [p22–28].

## Medical Education

**Frank Davidoff,** M.D., Editor Emeritus, *Annals of Internal Medicine*; Executive Editor for the Institute for Healthcare Improvement, USA, writes on, ‘Focus on Performance: The 21^st^ Century Revolution in Medical Education’ [p29–40].

## The Looking Glass

**Sander L. Gilman,** Ph.D., distinguished professor of the Liberal Arts and Sciences at Emory University, writes on,.Are Jews smarter than everyone else? [p41–47].

## Mental Health, Spirituality, Mind

**George E. Vaillant,** M.D. Professor of Psychiatry, Harvard Medical School, USA, writes on, ‘Positive emotions, spirituality and the practice of psychiatry’ [p48–62].

**E. Mohandas,** M.D. Chief Consultant Psychiatrist, Elite Mission Hospital, Kerala, India, writes on, ‘Neurobiology of spirituality’ [p63–80].

**Ajai R. Singh, M.D.** writes on,.Covert Treatment In Psychiatry: ‘Do No Harm, True, But Also Dare To Care’. [p81–109].

**William Hirstein,** PhD., Chair of the Philosophy Department at Elmhurst College, in Elmhurst, Illinois, writes on,.‘Mindmelding: Connected Brains and the Problem of Consciousness’. [p110–130].

## Musings

**L. Eugene Arnold,** M.Ed, M.D., Professor Emeritus of Psychiatry at Ohio State University, formerly director of the division of child and adolescent psychiatry and vice-chair of psychiatry, writes in *Musings*, ‘What child and adolescent psychiatry means to me’ [p131–134].

## Reflections

**Jayant V. Narlikar,** Founder Director, Inter-University Centre for Astronomy and Astrophysics (IUCAA), Past-President, Cosmology Commission of the International Astronomical Union, Astrophysicist and Science populariser, India, writes for *Reflections*, ‘Scientific research: What it means to me’ [p135–145].

## Ethical Issues in Biomedicine

**Leemon McHenry,** Ph.D., Lecturer in Philosophy, California State University, Northridge, USA, writes on, ‘Biomedical research and corporate interests: A question of academic freedom’ [p146–156].

**Murali Poduval** M.S., from the Department of Orthopedics, Seth G.S. Medical College and The KEM Group of Hospitals, Mumbai, India, and **Jayita Poduval,** M.S., ENT Surgeon, Thane district, Maharashtra, India, write on, ‘Medicine as a Corporate Enterprise: A Welcome Step?’ [p157–174].

## Poverty And Human Development

**Glenn D. Reeder,** PhD, and **John B. Pryor,** Ph.D., from the Illinois State University, USA, write on, ‘Dual psychological processes underlying public stigma and the implications for reducing stigma’ [p175–186].

**Ajai R. Singh,** M.D. **and Shakuntala A. Singh,** PhD., Editor and Deputy Editor respectively, *MSM*, write on, ‘Diseases of Poverty And Lifestyle, Well-being And Human Development’ [p187–225].

## Journalology

**John Hoey,** M.D., internist and public health physician, earlier editor of *CMAJ* (1996 to 2006), currently on the faculty at Queen.s University, Canada, involved in developing public health resources and teaching, writes on,.‘Editorial Independence In The Electronic Age: New Threats, Old Owners?’ [p226–236].

**Harvey Marcovitch,** M.D., earlier editor of *Archives of Disease in Childhood* (1994–2002), currently syndication editor for the BMJ Publishing Group and Associate Editor of the *BMJ*, and chairman of the Committee on Publication Ethics (COPE), writes in The Looking Glass on,.‘What medical journal editing means to me’ [p237–243].

**David Healy,** M.D., Professor of Psychiatry in Cardiff University, writes on, ‘Our Censored Journals’ [p244–256].

**Elise Langdon-Neuner,** Ph.D., Editor-In-Chief of The Write Stuff, journal of the European Medical Writers Association, Austria, writes on .Medical Ghostwriting [p257–273].

## MSM Book Review

**Ajit V. Bhide,** M.D., from the departments of psychiatry and family medicine at St. Martha's Hospital, Bangalore, writes a *MSM Book Review* of, ‘*Oxford Textbook Of Philosophy And Psychiatry*’ (by K.W.M. Fulford, T. Thornton, and G. Graham, Oxford University Press, New York, 2006. Indian edition, OUP New Delhi, 2007) [p274–276].

## MSM Poems

**Kristina Brenner,** currently finishing a Masters of Public Health degree, writes for *MSM Poems*, ‘Haiku: Philosophy, metaphysics’, and, ‘The Body of Science’ [p277–279].

## Readers Respond

**N. N. Wig,** Prof. Emeritus Psychiatry, PGIMER, Chandigarh, India, responds [p280].

## Dedication

This issue of MSM is dedicated to the fond memory of Prof N.S. Vahia. See Dedication by the **Editor** [pxii–xiv].

## Obituary

**Daniel Raveh,** Ph.D., lecturer, Department of Philosophy, Tel-Aviv University who studied under and worked with Prof. Daya Krishna for the last decade writes an obituary, ‘Prof Daya Krishna (1913–2007)’ [p281–284].

## Honorary International Advisory Editorial Board Members

Read about the board members in short [p1-2] and their bios [p285–292].

## Call for Papers

See Call for Papers MSM 2009 on Women's Issues [p*xv*] and MSM 2010 on Psychopharmacology Today [p*xvii*]

